# 96 A Quality Improvement Study of Routine Mattress Exchange to Reduce Microbial Recontamination

**DOI:** 10.1093/jbcr/irad045.069

**Published:** 2023-05-15

**Authors:** W Michael Woolfolk, Patrick Brockway, Kenneth Mouser, Tina Boam, Kais Atmeh, Nicole Lemieux, J Tracy Anderson, Sai Velamuri, Xiangxia Liu, David Hill

**Affiliations:** Firefighters Burn Center, Memphis, Tennessee; Firefighters Burn Center, Memphis, Tennessee; Firefighters Burn Center, Memphis, Tennessee; Firefighters Burn Center, Memphis, Tennessee; UTHSC, Memphis, Tennessee; Regional One Health, Memphis, Tennessee; Firefighters Burn Center, Memphis, Tennessee; University of Tennessee, Memphis, Tennessee; Firefighters Burn Center, Memphis, Tennessee; Regional One Health, Memphis, Tennessee; Firefighters Burn Center, Memphis, Tennessee; Firefighters Burn Center, Memphis, Tennessee; Firefighters Burn Center, Memphis, Tennessee; Firefighters Burn Center, Memphis, Tennessee; UTHSC, Memphis, Tennessee; Regional One Health, Memphis, Tennessee; Firefighters Burn Center, Memphis, Tennessee; University of Tennessee, Memphis, Tennessee; Firefighters Burn Center, Memphis, Tennessee; Regional One Health, Memphis, Tennessee; Firefighters Burn Center, Memphis, Tennessee; Firefighters Burn Center, Memphis, Tennessee; Firefighters Burn Center, Memphis, Tennessee; Firefighters Burn Center, Memphis, Tennessee; UTHSC, Memphis, Tennessee; Regional One Health, Memphis, Tennessee; Firefighters Burn Center, Memphis, Tennessee; University of Tennessee, Memphis, Tennessee; Firefighters Burn Center, Memphis, Tennessee; Regional One Health, Memphis, Tennessee; Firefighters Burn Center, Memphis, Tennessee; Firefighters Burn Center, Memphis, Tennessee; Firefighters Burn Center, Memphis, Tennessee; Firefighters Burn Center, Memphis, Tennessee; UTHSC, Memphis, Tennessee; Regional One Health, Memphis, Tennessee; Firefighters Burn Center, Memphis, Tennessee; University of Tennessee, Memphis, Tennessee; Firefighters Burn Center, Memphis, Tennessee; Regional One Health, Memphis, Tennessee; Firefighters Burn Center, Memphis, Tennessee; Firefighters Burn Center, Memphis, Tennessee; Firefighters Burn Center, Memphis, Tennessee; Firefighters Burn Center, Memphis, Tennessee; UTHSC, Memphis, Tennessee; Regional One Health, Memphis, Tennessee; Firefighters Burn Center, Memphis, Tennessee; University of Tennessee, Memphis, Tennessee; Firefighters Burn Center, Memphis, Tennessee; Regional One Health, Memphis, Tennessee; Firefighters Burn Center, Memphis, Tennessee; Firefighters Burn Center, Memphis, Tennessee; Firefighters Burn Center, Memphis, Tennessee; Firefighters Burn Center, Memphis, Tennessee; UTHSC, Memphis, Tennessee; Regional One Health, Memphis, Tennessee; Firefighters Burn Center, Memphis, Tennessee; University of Tennessee, Memphis, Tennessee; Firefighters Burn Center, Memphis, Tennessee; Regional One Health, Memphis, Tennessee; Firefighters Burn Center, Memphis, Tennessee; Firefighters Burn Center, Memphis, Tennessee; Firefighters Burn Center, Memphis, Tennessee; Firefighters Burn Center, Memphis, Tennessee; UTHSC, Memphis, Tennessee; Regional One Health, Memphis, Tennessee; Firefighters Burn Center, Memphis, Tennessee; University of Tennessee, Memphis, Tennessee; Firefighters Burn Center, Memphis, Tennessee; Regional One Health, Memphis, Tennessee; Firefighters Burn Center, Memphis, Tennessee; Firefighters Burn Center, Memphis, Tennessee; Firefighters Burn Center, Memphis, Tennessee; Firefighters Burn Center, Memphis, Tennessee; UTHSC, Memphis, Tennessee; Regional One Health, Memphis, Tennessee; Firefighters Burn Center, Memphis, Tennessee; University of Tennessee, Memphis, Tennessee; Firefighters Burn Center, Memphis, Tennessee; Regional One Health, Memphis, Tennessee; Firefighters Burn Center, Memphis, Tennessee; Firefighters Burn Center, Memphis, Tennessee; Firefighters Burn Center, Memphis, Tennessee; Firefighters Burn Center, Memphis, Tennessee; UTHSC, Memphis, Tennessee; Regional One Health, Memphis, Tennessee; Firefighters Burn Center, Memphis, Tennessee; University of Tennessee, Memphis, Tennessee; Firefighters Burn Center, Memphis, Tennessee; Regional One Health, Memphis, Tennessee; Firefighters Burn Center, Memphis, Tennessee; Firefighters Burn Center, Memphis, Tennessee; Firefighters Burn Center, Memphis, Tennessee; Firefighters Burn Center, Memphis, Tennessee; UTHSC, Memphis, Tennessee; Regional One Health, Memphis, Tennessee; Firefighters Burn Center, Memphis, Tennessee; University of Tennessee, Memphis, Tennessee; Firefighters Burn Center, Memphis, Tennessee; Regional One Health, Memphis, Tennessee

## Abstract

**Introduction:**

It is industry standard for a patient to maintain a single mattress throughout admission, which may be problematic with burn injuries. Significant burn injuries are high exudate wounds which provide challenging care, including frequent dressing changes and environmental contamination. As part of an infection prevention process improvement (PI) including routine air mattress (AM) exchanges throughout admission, the purpose of this study was to examine microbial contamination of mattresses before and after utilization.

**Methods:**

This single center, retrospective study was conducted in an ABA-verified burn center. Routine AM exchanges began in November 8, 2020, including logging serial numbers of incoming/outgoing AM and periodic environmental cultures. Pre- and post-occupation AM environmental cultures (EC) (outside and inside the top cover and baffles) were compared with patient culture data. All patients were admitted to the Burn Center.

**Results:**

During the 91 weeks, a total of 635 AM were exchanged. A total of 34 AM were cultured with 20 (59%) being post- and 14 being the pre-occupation. For the pre-occupation AM cultures, all 14 outer and inner surfaces cultured sterile. Regarding post-occupation AM, 80% had a positive EC of the outside surface, while 58% had positive environmental culture from inside the sealed AM. The median time of AM utilization prior to culturing was 6 (6, 7) days. All microbes isolated from post-occupation AM matched culture data from each patient’s hospital course. There were a total of 42 microbes isolated from environmental swabs taken from the outside cover with the most common: S.epidermidis 38%, S.aureus 14%, and P.aeruginosa 12%. A total of 40 microbes were isolated from inside the sealed AM with the most common: S.epidermidis 25%, Acinetobacter spp. 13%, Enterobacter spp. 10%, and Pseudomonas spp. 10%. There were 5 different EC taken from inside the sealed AM resulting in fungal growth. The 34 EC were taken from AM of 9 patients during the 91 week period. The median age was 48 (22, 66) years with 42 (21.25, 44) %TBSA burned. The median weight was 92 (76, 98) kg with body mass index of 27.47 (23.72, 37.11) kg/m2. Two of the 9 patients were below 20 % TBSA burned (9 and 19.5%) while the remaining 7 patients had greater that 20% TBSA burns. There was an inverse signals between size of burn and amount of wound closure and contamination.

**Conclusions:**

AM are not impervious to the heavy exudate of patients with large burn injuries. Over time, the mattress becomes contaminated with microorganisms specific to the patient. This study justifies the infection prevention practice of routine mattress exchanges during admission for patients with large, open burn wounds. We do not send patients back from the operatory theater or hydrotherapy with reused dressing. Given these findings mattresses should be no different.

**Applicability of Research to Practice:**

First study to analyze infectious mitigation potential of routine AM exchanges in burn patients.

